# Genetics and genomic medicine in Sri Lanka

**DOI:** 10.1002/mgg3.744

**Published:** 2019-05-20

**Authors:** Nirmala D. Sirisena, Vajira H. W. Dissanayake

**Affiliations:** ^1^ Human Genetics Unit, Faculty of Medicine University of Colombo Sri Lanka

**Keywords:** clinical genetics, genetic testing, genomic medicine, genomics, medical genetics

## Abstract

The completion of the Human Genome Project in 2003 heralded in a new era marked by remarkable advances in biomedical research leading to the establishment of genomics‐based translational medicine mainly in the developed world. However, the development of such advances has been hampered in most parts of the developing world due to scarcity of resources and trained personnel. Genetics and genomic medicine are currently in the process of being integrated into the Sri Lankan health care system. These developments have taken place mainly due to the heightened awareness and increasing demands made by the public for provision of genetic diagnostic and therapeutic services in clinical care. Due to the exorbitant costs incurred in the maintenance of these services and the dearth of adequately trained manpower, only a few centers in the country, mainly in Universities or private sector, are currently engaged in providing these services to the public. This article aims to provide an overview of the genetics and genomic medicine services in Sri Lanka from its early developments to the current state.

## 1 INTRODUCTION

### 1.1 Geographic and sociodemographic data

**Figure 1:**
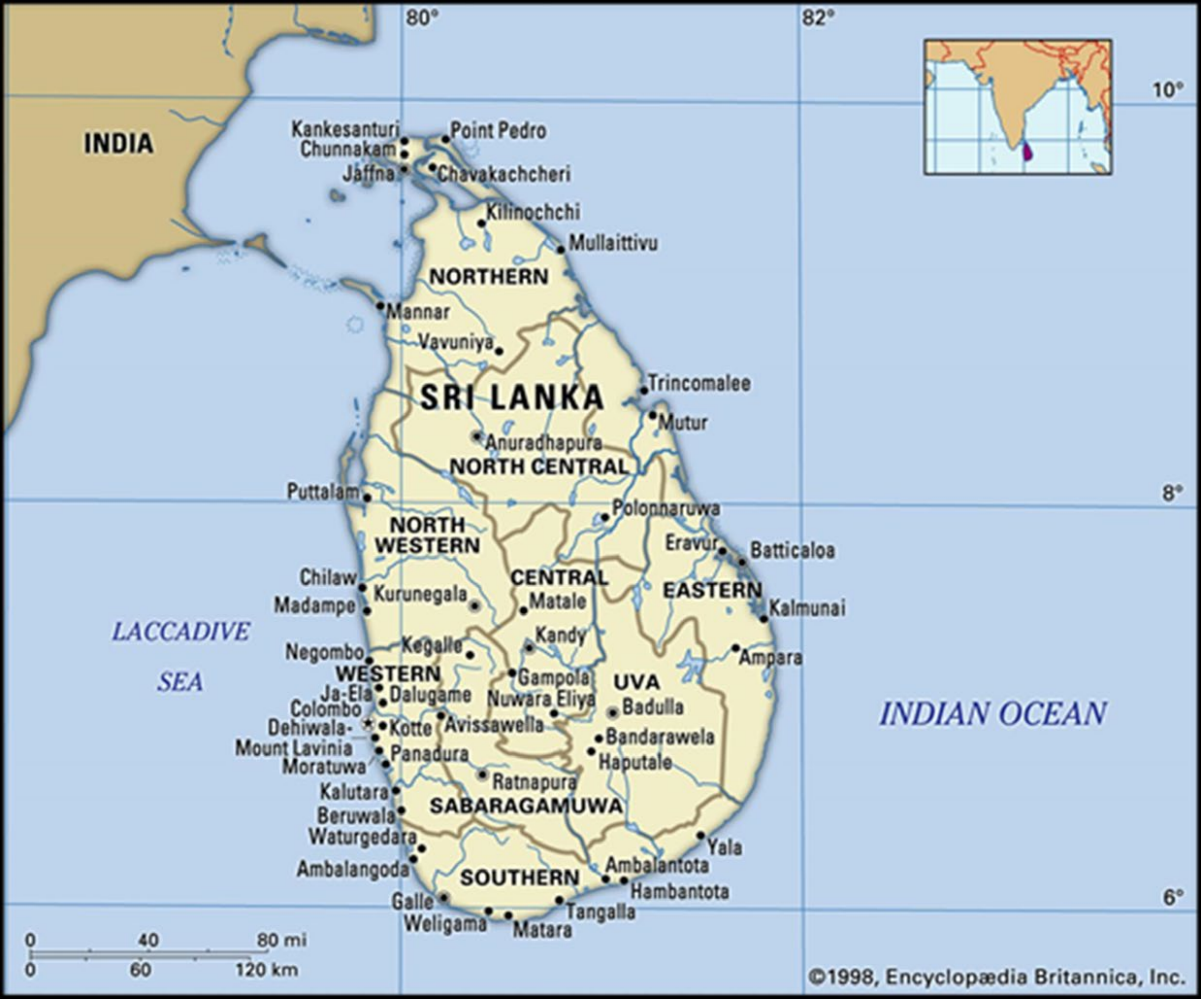
Political map of Sri Lanka (Adapted from Encyclopaedia Britannica—Peiris, 2019)

Sri Lanka, formerly known as Ceylon, is an island nation in the Indian Ocean. It is located between latitudes 5°55′ and 9°51′ North and longitudes 79°41′ and 81°53′ East and has a maximum length of 432 km and a maximum width of 224 km with a total land area of 65,610 km^2^ (Peiris, 2019). The executive and judicial capital of Sri Lanka is Colombo while Sri Jayewardenepura Kotte is the legislative capital. The country is divided into nine provinces (Northern, North Central, North Western, Central, Western, Eastern, Southern, Sabaragamuwa, and Uva) which are further subdivided into 25 districts for administrative purposes (Figure 1). Thalassemia, which is the commonest single‐gene disorder in the country, tends to cluster in the North Western, North Central, Central, and Uva provinces (De Silva, 2004).

**Figure 2:**
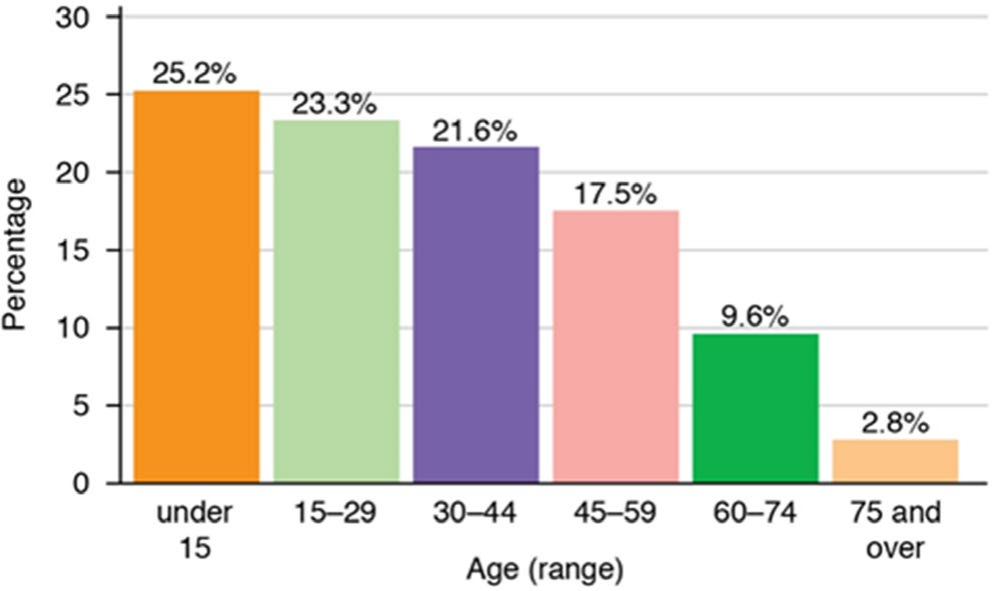
Age structure of the Sri Lankan population (2016) (Adapted from Encyclopaedia Britannica—Peiris, 2019)

As at 2016, the mid‐year population of Sri Lanka was 21,688,000 with a population density of 338 persons/km^2^. Around 81.5% of the Sri Lankan population live in rural areas and only about 18.5% live in the urban areas. Figure 2 shows that around 70% of the Sri Lankan population are aged under 45 years. Sri Lanka is a culturally and linguistically diverse nation.

Sinhalese, Sri Lankan Tamils, Indian Tamils, and Moors are the ethnic groups which constitute more than 99% of the Sri Lankan population. The Sinhalese are an indigenous population that according to legend intermarried with migrants from the Bengali region of India over 2,500 years ago. They constitute almost 75% of the population. The Tamils comprise of two groups—Sri Lankan Tamils who are descendants of early settlers from southeastern India accounting for around 12.5% of the total population and Indian Tamils who are recent immigrants from southern India who constitute about 5.5% of the total population. Moors are descendants of Arab traders who intermarried with the indigenous women and they account for about 7.5% of the population. Burghers, who comprise a group of mixed European descent; Malays, who are immigrants from eastern Asia; and Veddas, who are considered as the indigenous inhabitants of the country, together account for less than 1% of the total population. Paucity of data exists about the population genetics of the major Sri Lankan ethnic groups as no large‐scale projects have been undertaken to catalog the allele frequencies of single nucleotide variants in the population (Chan et al., 2016; Sirisena & Dissanayake, 2017).

Religious affiliations indicate that Buddhists comprise almost 70.1% of the total population while the rest are Hindus (12.6%), Muslims (9.7%), Roman Catholics (6.2%), and Christians (1.4%). The literacy rate of the population aged above 15 years is 96.8% for males and 94.6% for females. As of 2016, the gross national income (GNI) per capita amounted to 3,835 US dollars and Sri Lanka belonged to the lower middle‐income country category according to the World Bank classification. The expenditure on education constitutes about 5.4% of the GNI while health expenditure accounts for about 1.5% of the GNI (Department of Census & Statistics, 2019; Peiris, 2019).

### 1.2 Health care services and health indicators in Sri Lanka

A comprehensive public sector national health service funded by the government through taxation delivers free health care services throughout the country through a broad network of hospitals and other health care institutions. A wide range of preventive health care programs as well as a well‐organized maternal and child health program have contributed substantially to improving the health conditions of the Sri Lankan population with health indicators on par with the developed world in spite of low investment in healthcare. A smaller private sector in Western medicine coexists alongside the public health services. Several indigenous traditional medicine healthcare systems—Ayurveda, Unani, Sidda—also contribute significantly to Sri Lankan medical practices (Peiris, 2019).

As of 2015, the number of live births in Sri Lanka was 334,821 with an infant mortality rate of 8.5 per 1,000 live births, an under‐5 mortality rate of 10.1 per 1,000 live births and a maternal mortality ratio of 25.7 per 100,000 live births. More than 99.9% of births occur in hospitals and 15% of births are by women aged above 35 years (Department of Census & Statistics, 2019). The major health problems in the pediatric population include malnutrition, various infectious diseases, and birth defects. Life expectancy at birth for males is 73.3 years and 80.4 years for females (Department of Census & Statistics, 2019; Peiris, 2019).

### 1.3 History of medical genetics and genomics in Sri Lanka

The birth of modern genetics is traced back to the work of Gregor Mendel in the 19th century. However, the effect of genes on human health and wellbeing had been well known to the Eastern civilizations several centuries earlier. The ancient Buddhist scriptures refer to the “laws of the seeds.” Archeological finds among the ruins of the ancient capital Anuradhapura in Sri Lanka dating back to the 4th century AD show achondroplastic dwarfs carved into the guardstones of Buddhist temples (Godakumbura, 1969). Although the outwardly recognizable birth defects were identified from ancient times, evidence to support the genetic basis of these conditions was found only after the development of modern science. On July 18, 1960, George W. Corner stated in his theme address to the First International Conference on Congenital Malformations that "at least one type of congenital malformations in human beings—Mongolism—can now be definitely ascribed to a genetic mechanism" (Corner, 2019).

Before the dawn of genetic testing, the birth of a child with a congenital anomaly was the primary factor that drew attention to genetics. The earliest report of a congenital anomaly in the Sri Lankan scientific literature was a case report of an imperforate anus written by Hallock in the inaugural volume of the Ceylon Medical Journal published in 1888 (Hallock, 1888). In 1938, Misso and Sandrasagar reported the anatomical abnormalities observed in the dissecting room of the Ceylon Medical College (Misso & Sandrasagar, 1938). Almost four decades later in 1982, Corea documented the Epidemiology of Congenital Malformations in Sri Lanka in the MD thesis to the Postgraduate Institute of Medicine (PGIM), Colombo (Corea, 2019). This is by far the earliest and the most comprehensive documentation of such conditions in the country.

The formal origin of medical genetics in Sri Lanka dates back to the 1960s and 1970s (Dissanayake, 2012). The first Sri Lankan doctor to train formally in Human Genetics was Prof. Eugene Wickramanayake. She was a member of the academic staff of the Department of Anatomy at the Peradeniya Campus of the University of Ceylon. Dr. Wickrmanayake trained at the University of Edinburgh, UK and returned back home in 1968. She started Barr body testing in a laboratory established in her department and started an introductory course in genetics for undergraduates. She participated in the Fourth International Congress of Human Genetics in Paris in 1971 where she presented a paper. She had a special interest in the genetics of Veddas and together with her colleague Dr. S. B. Ellepola, who later became Professor of Pathology at the same Faculty, she carried out population genetic studies on Uva Bintenna Veddas. Dr. Ellepola's MD thesis in 1985 was titled “A genetics study of Veddas of Sri Lanka” (Ellepola, 2019).

The credit of establishing Human Genetics as a separate specialty in the Faculty of Medicine, University of Colombo goes to Prof. Rohan W. Jayasekara, Chair and Senior Professor of Anatomy and Medical Geneticist from 1999 to 2015. Dr. Jayasekara returned back home in 1981 after his training in Human Genetics at the University of Newcastle upon Tyne, UK. He founded the Human Genetics Unit (HGU) in the Faculty in October 1983 with support from the World Health Organization and served as its Director until August 2014. This historic endeavor marked the first major initiative undertaken by a Sri Lankan academic towards the provision of clinical genetics and genetic diagnostic services in the country. The HGU now serves as the national referral center in Sri Lanka, serving a population of 21 million people and is dedicated to providing clinical genetics, genetic diagnostics and genomic medicine services, as well as genetics education and research.

### 1.4 Genetic diagnostic services

Cytogenetic testing as a routine laboratory diagnostic service was introduced at the HGU in 1983 by Prof. Jayasekara. In the later part of the 1980s when molecular diagnostics came into clinical practice, he collaborated with his colleagues in the Department of Biochemistry, who had by then established polymerase chain reaction (PCR) technology for the first time in Sri Lanka in their laboratory (Yasaratne, 2019), to conduct what was the first molecular genetic study investigating the etiology of a medical condition (Duchenne muscular dystrophy) in Sri Lanka (Welihinda et al., 1993).

In 1998, the senior author of this paper joined the HGU at a time when genetics was not a highly sought‐after specialty by medical professionals in the country. After completing his doctorate training at the University of Nottingham, UK, he returned back home in 2004, and together with Prof. Jayasekara, embarked on expanding genetic services both in the University sector as well as in the private sector. In the decade that followed, the following genetic diagnostic services were introduced to the country: automated karyotyping (2005); PCR‐based molecular genetic testing (2006); Sanger sequencing using an Applied Biosystems Capillary Sequencer (2006); fluorescence in situ hybridization (2012); and next‐generation sequencing (NGS; 2014). Aside from the HGU, another public sector academic unit that has been established includes the Molecular Medicine Unit at the Faculty of Medicine, University of Kelaniya which specializes in infectious diseases diagnostics and DNA typing for human identification and forensic case work. The same Faculty has a specialized unit dedicated to diagnosing and treatment of patients with thalassemia.

**Figure 3:**
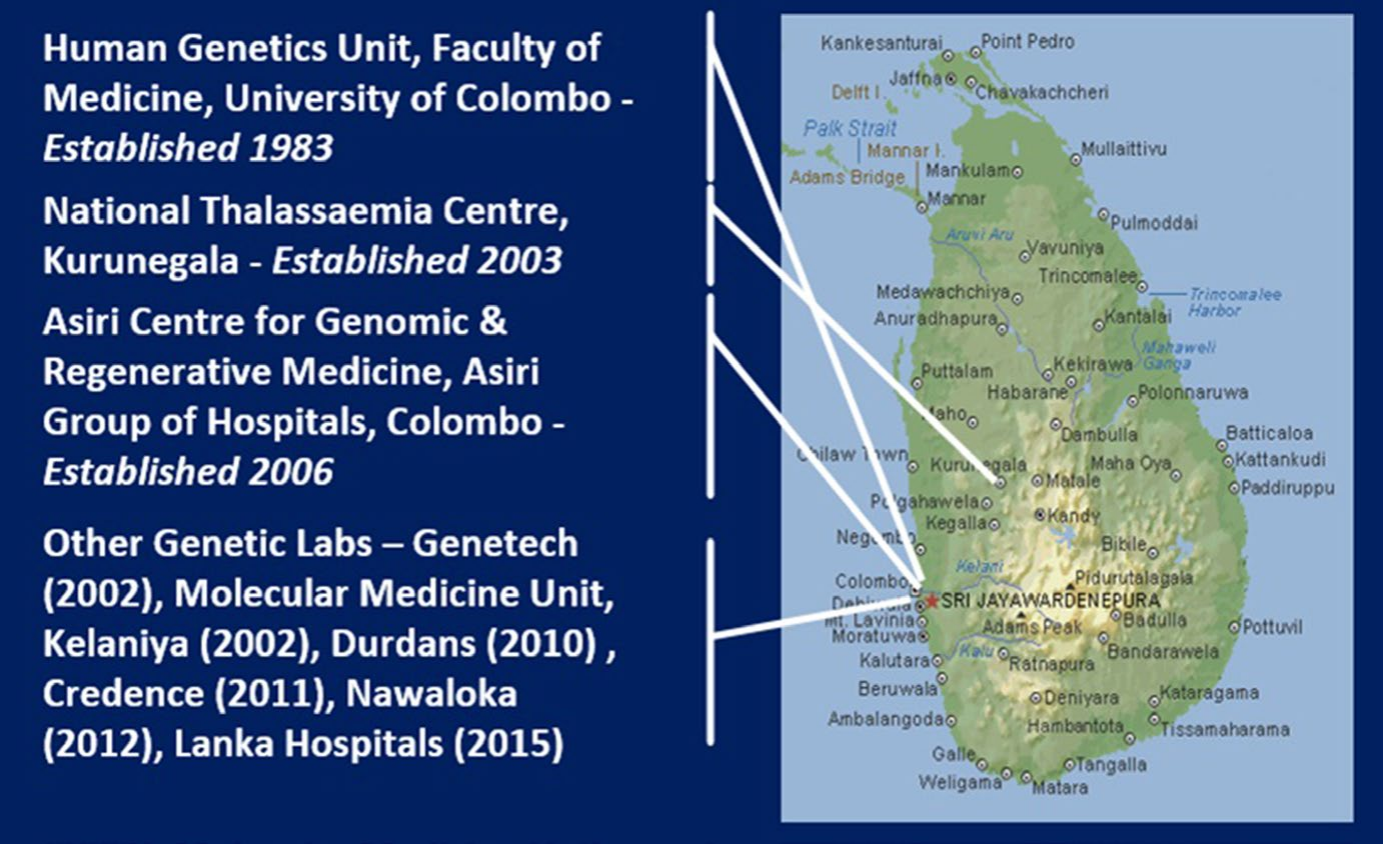
Distribution of genetic diagnostic centers in Sri Lanka

In addition to these developments in the public sector, genetic diagnostic services have grown in the private sector as well. The first private sector laboratory for genetics was the Genetech laboratory [http://www.genetechsrilanka.com] setup in 2002 by Dr. Maya Gunasekara, a scientist. Some of the services offered by the laboratory include DNA fingerprinting and paternity testing. By far, the most comprehensive private sector genetic diagnostics service in the country today is provided by the Asiri Center for Genomic and Regenerative Medicine (ACGRM) [http://www.asiri.lk/acgrm] established in 2006 in the Asiri Surgical Hospital by the senior author of this paper. It provides cytogenetic and molecular genetic diagnostics for a wide range of genetic disorders and infectious diseases including genetic counseling services and prenatal diagnosis (Dissanayake, 2012). Since then, genetic diagnostics laboratories have been established in several other private hospitals in Colombo such as the Durdans Hospital, Nawaloka Hospital, Lanka Hospital and Credence Genomics (Figure 3).

### 1.5 Clinical genetics and genetic counseling services

The first routine clinical genetics services in the country were started by Prof. Jayasekara at the HGU in 1981. His genetics clinics were very popular and patients were referred from all parts of the country for genetic evaluation and counseling (Jayasekara, Kristl, & Wertelecki, 1988). He ensured that these services were introduced to the country while carefully taking into consideration the cultural sensitivities specific to the Sri Lankan context (Jayasekara, 1989). Several decades later, in 2007, the HGU, started offering telegenetics services to enable rural communities to access genetic counseling (Dissanayake, Nisansala, Sandamal, & Jayasekara, 2010). This was one of the first such services anywhere in the developing world. This service, however, was difficult to sustain because in Sri Lanka people have ready access to health care facilities where they can meet a doctor face to face. The HGU has continued to be in the forefront of adopting information and communication technology and has embarked on the process of introducing electronic medical records. The HGU was also one of the first academic or clinical centers in Sri Lanka to have its own website almost 20 years ago [http://www.hgucolombo.org]. Various subspecialty clinics dedicated to providing clinical genetics and genetic counseling services are now available at the Unit. These include: dysmorphology, reproductive genetics, cancer genetics, hematology and hemato‐oncology, neurogenetics, and ophthalmogenetics.

### 1.6 Implementation of genomic medicine in Sri Lanka

In this background, in 2010, Sri Lanka entered the genomics era with the sequencing of the complete genome of a Sri Lankan. In 2014, using the Illumina MiSeq NGS platform and an in‐house developed bioinformatics pipeline using open‐source software, the HGU successfully implemented clinical exome sequencing, whole exome sequencing, and multigene cancer panel testing to the repertoire of services that are offered. Today genomic medicine services have become a routine part of the services offered by the HGU (Sirisena & Dissanayake, 2018; Sirisena, Neththikumara, Wetthasinghe, & Dissanayake, 2016; Sirisena, Sumathipala, Wettasinghe, & Dissanayake, 2016). The implementation of genomic medicine into routine clinical practice has facilitated improved care for Sri Lankan patients by enabling Sri Lankan doctors to diagnose and successfully manage patients with a spectrum of genetically heterogeneous rare disorders with unusual coexisting phenotypes and inherited cancer syndromes, all of whom hitherto lacked a precise genetic diagnosis and appropriate treatment. It has also enabled us to build a catalogue of genetic variants in the Sri Lankan population. The progressive expansion of clinical genetic and genetic diagnostic services, education, and research at the HGU has made it one of the leading centers for genetics and genomics in the developing world.

### 1.7 Education and training in medical genetics and genomic medicine

The introduction of basic medical genetics and clinical genetics to the undergraduate medical curriculum at the Medical Faculties in Universities of Colombo, Kelaniya, and Sri Jayewardenepura as well as to postgraduate medical courses in over a dozen medical specialty courses conducted by the PGIM [http://www.cmb.ac.lk/pgim/] of the University of Colombo, which trains all medical specialists in the country, was initiated by Prof. Jayasekara. At present, basic medical genetics is a part of the basic sciences stream curriculum in all medical schools in the country. Medical genetics is a part of the curriculum in Surgery, Medicine, Obstetrics and Gynecology, Pediatrics, Family Medicine, Transfusion Medicine, Hematology, Oncology and Radiotherapy, Molecular Medicine, Community Dentistry, Legal Medicine and Forensic Science, Medical Administration, and Sports Medicine.

The HGU took steps to further expand genetics education by starting Masters Courses in Clinical Genetics and Genetic Diagnostics in 2010 in collaboration with the University of Oslo, Norway. A Masters course in Stem Cell Biology and Regenerative Medicine was started in 2012 in collaboration with the Manipal University, India. These Masters courses have so far produced 16 clinical geneticists and 21 scientists. A Masters course in Molecular Pathology was introduced in 2018. The first PhD program in Human Genetics was started in the HGU in 2006. This program has so far produced eight academic scientists some of whom are now working in three Universities in the country. As a result of creation of this manpower, clinical genetics services have now been extended to other parts of the country and specialist genetics clinics have been established in institutions such as the Children's Hospitals in Colombo and Peradeniya; the National Cancer Institute in Maharagama, and the National Eye Hospital in Colombo. In addition, continuing medical education courses in genetics and genomics for medical and allied health professionals, as well as programs aimed at raising genetic awareness among the public are also being conducted in collaboration with other professional medical colleges and associations in the country.

### 1.8 Research in medical genetics and genomic medicine

It is interesting to note that the first report of a single gene disorder in the Sri Lankan scientific literature was published in 1938. That was a paper on color blindness by Prof. F.O.B. Ellison, the Professor of Physiology at the Colombo Medical School (Ellison, 1938). The first paper documenting chromosome anomalies in Sri Lanka was published in 1988 by Prof. Jayasekara (Jayasekara, 1988). The first paper on the commonest single gene disorder in the country, thalassemia, was published by the Professor of Pediatrics at the Faculty of Medicine, University of Ceylon, Prof. C. C. De Silva in 1957 (De Silva, 1957).

The HGU has research groups in clinical genetics, cytogenetics, cancer genetics and genomics, reproductive genetics, regenerative medicine and stem cell biology, and bioinformatics. These groups have generated over 350 million rupees in grants and produced over 90 research papers and 250 conference abstracts in the past decade. The Unit as well as its team have been recognized for their work with awards at international, national, and University levels as well as at many international and local conferences. The current research activities of the Unit are geared toward identifying the spectrum of genomic variations specific to the Sri Lankan population in inherited cancer syndromes, myelodysplastic syndromes, and rare genetic disorders. As a genetic center of excellence, the future goals of the HGU are to increase our understanding of the genetic architecture of the Sri Lankan population, to enhance our bioinformatics capabilities to enable robust support to the clinical genetics services, identification of novel therapeutic targets, and develop novel therapies tapping in to the biodiversity of Sri Lanka.

### 1.9 Pattern of genetic diseases in Sri Lanka

Chromosomal disorders, rare single gene disorders, and inherited cancer syndromes constitute the major genetic disorders seen in the Sri Lankan population. Although antenatal genetic testing for chromosomal abnormalities and other rare disorders are currently available, many children with these disorders are born because termination of pregnancies for a fetal indication is legally prohibited in the country. A retrospective audit of the cytogenetic reports of 1,554 consecutive children with suspected chromosomal disorders who underwent karyotyping at the two main genetic diagnostic centers (HGU and ACGRM) from January 2006 to December 2011 showed that abnormal karyotypes were found in 783 (50.6%) children (Thillainathan, Sirisena, Kariyawasam, Jayasekara, & Dissanayake, 2015). Numerical and structural abnormalities accounted for 90.8% and 9.2%, respectively. Down syndrome (84.9%) was the most common disorder identified, followed by Turner syndrome (6.4%) and Edward syndrome (2.3%). A similar audit conducted at the same two centers on 338 women with primary amenorrhea referred for cytogenetic analysis from January 2005 to December 2011 showed that numerical and structural chromosomal abnormalities were detected in 115 (34.0%) patients (Samarakoon et al., 2013). The most common disorders included 45, X Turner syndrome (10.7%), Turner syndrome variants (13.9%), and XY females (6.5%) due to androgen insensitivity syndrome. An audit of 213 couples and 16 individuals referred for cytogenetic analysis of subfertility and recurrent pregnancy losses showed that chromosomal abnormalities and polymorphic variants that could interfere with fertility were found in 9.5% of the patients (Dissanayake, Athapaththu, et al., 2010).

Thalassemia is by far the most common single‐gene disorder in Sri Lanka with the beta‐type being more predominant than the alpha‐type. It is estimated that there are approximately 2,000 thalassemia patients in the country who consume almost 7.5% of the national health budget per annum for transfusions and iron‐chelation costs (Dissanayake, 2012). The commonest beta‐globin gene (*HBB*; OMIM 141,900; NM_000518.5) variants which account for greater than 85% of beta‐thalassemia in the country include: IVSI‐5 (G > C); IVSI‐1 (G > A); codon (CD) 26 (G > A); CD 6 (−13 bp); IVSI‐129 (A > C), and CD 55 (−A) (Fisher et al., 2003). In contrast, alpha‐thalassemia is mainly due −α^3·7^ and −α^4·2^ deletions in the alpha‐globin genes: hemoglobin subunit alpha 1 (*HBA1*; OMIM 141800; NM_000558.5) or hemoglobin subunit alpha 2 (*HBA2*; OMIM 141850; NM_000517.6) (De Silva et al., 2000). Other common monogenic disorders include: hemophilia A, neurodegenerative disorders such as Duchenne muscular dystrophy, Huntington's disease, and spinocerebellar ataxias (Dissanayake, 2012). By and large, the implementation of NGS services at the HGU has led to the unraveling of the genetic etiology of a plethora of genetically heterogeneous rare disorders and inherited cancer syndromes (Hettiarachchi, Nethikumara, et al., 2018; Hettiarachchi, Neththikumara, Pathirana, Padeniya, & Dissanayake, 2018; Sirisena, Neththikumara, et al., 2016).

### 1.10 The national birth defects surveillance program

Even though Sri Lanka has lower levels of infant mortality, birth defects assume a major cause of the infant and under‐5 mortality. Preliminary data suggest that birth defects may account for about 26% of these deaths. It is estimated that around 5,800 children are born each year with birth defects and about 30% of them have severe anomalies which prevent them from living an independent life. The major causes of birth defects include: congenital heart anomalies (24.0%), limb anomalies (20.3%), cleft lip and palate (8.5%), chromosomal abnormalities (6.7%), and neural tube defects (5.9%) (Unpublished data). Several strategies are now in place for the prevention and control of birth defects. Due to the unavailability of quality birth defects data in the country, the Ministry of Health has recently introduced a birth defects surveillance mechanism, with Family Health Bureau, as the national nodal point.

### 1.11 Pharmacogenomics

The allelic diversity of more than 7,000 genetic variants involved in drug biotransformation and response in the three major ethnic populations of Sri Lanka (Sinhalese, Sri Lankan Tamils, and Moors), were compared with other South Asian, South East Asian, and European populations (Chan et al., 2016). Overall, high levels of similarity within the Sri Lankan populations and between Sri Lankan and other South Asian populations were observed but substantial differences between Sri Lankan and European populations for important pharmacogenomic variants related to warfarin [Vitamin K epoxide reductase complex subunit‐1 (*VKORC1*) (OMIM 608547) (NM_024006.5:c.‐1639G>T), rs9923231] and clopidogrel [cytochrome P450 family 2 subfamily C member 19 (*CYP2C19*) (OMIM 609535) [NM_000769.1:c.636G>A (p.Trp212Ter)], rs4986893] were reported (Chan et al., 2016). Another study which investigated genetic variants in the cytochrome P450 family 2 subfamily D member 6 gene (*CYP2D6*) (OMIM 608902) [NM_000106.5:c.454delT (p.Trp152Glyfs), rs5030655] in the Sri Lankan population showed that CYP2D6*3 [NM_000106.5:c.775delA (p.Arg259Glyfs), rs35742686], CYP2D6*4 [NM_000106.5:c.506‐1G>A, rs3892097], and CYP2D6*10 [NM_000106.6:c.100C>T (p.Pro34Ser), rs1065852] variants, which are associated with reduced or loss of *CYP2D6* enzyme function were found in our population in significant frequencies (Tharanga et al., 2013). Genetic diagnostic assays for detection of activating mutations in the V‐Ki‐Ras2 Kirsten rat sarcoma 2 viral oncogene homolog (*KRAS*) [OMIM 190070] [NM_004985.4] and B‐Raf proto‐oncogene, serine/threonine kinase (*BRAF*) [OMIM 164757] [NM_004333.4] genes in patients with metastatic colorectal cancer (mCRC) are currently available in the country.

An audit of the *KRAS* genotypes of 108 colorectal tissue samples tested at ACGRM showed that *KRAS* mutations were present in 25 (23.0%) samples. Among the *KRAS*‐positive cases, 60.0% had point mutations in codon 12 and 40.0% had a single mutation in codon 13. These assays have aided clinicians in selecting appropriate patients with mCRC for anti‐EGFR therapy, thereby avoiding unnecessary adverse effects and minimizing treatment costs (Sirisena, Deen, Mandawala, Herath, & Dissanayake, 2017).

### 1.12 Global initiatives

Sri Lanka has continued to play a significant role in genetics and genomics in the international arena as well. The first team of doctors and scientists trained in medical genetics in Nepal were graduates of the Masters courses conducted at the HGU. The first Medical Genetics Unit in Nepal was established with this team consisting of a clinical geneticist and two scientists at the National Academy of Medical Sciences in Kathmandu, Nepal in 2015. Further, the senior author of this paper was among the invitees to the first ever Meeting of Global Leaders in Genomic Medicine convened by the Institute of Medicine of the National Academy of Sciences of USA in Washington DC in January 2014. This led to the establishment of the Global Genomic Medicine Collaborative (G2MC) of which he is one of four founding Executive Board Members. G2MC is spearheading genomic medicine adoption across the world with a focus on the developing world (Manolio et al., 2015).

## 2 CONCLUSIONS AND FUTURE PROSPECTS

From its early days, medical genetics and genomics has come a long way in Sri Lanka and has now been recognized by the Ministry of Health in its strategic plan from 2016 to 2025 as a specialty that needs to be developed in the country. The preliminary steps have already been taken to formally recognize clinical genetics as a medical specialty with board certification as well as to produce board certified pathologists specializing in laboratory genetics. Plans are in the pipeline to increase medical genetics literacy among practicing health care professionals by introducing continuing professional development programs based on a curriculum of core competencies in medical genetics and genomic medicine with optional modules for each subspecialty area. Integrating medical genetics into the primary health care services and improving accessibility to genetic diagnostics and counseling services outside of Colombo are matters that need to be further addressed. In this background, it is necessary to dwell on establishing the ethical framework for the implementation of these services in the country. The background for this was laid by a study group convened by the National Science and Technology Commission of Sri Lanka in 2003 chaired by Prof. Jayasekara (NASTEC, 2019). Advocacy to make genetic services freely available to Sri Lankans under the national health services and contribution to policy formation in the field of genetics are constantly being addressed. These developments will lay a firm foundation for further development and expansion of medical genetics and genomic services in Sri Lanka in the coming years.

## CONFLICT OF INTEREST

The authors declare that they have no conflicts of interest.
